# Assessment of ecological impairment of Arctic streams: Challenges and future directions

**DOI:** 10.1002/ece3.7798

**Published:** 2021-06-26

**Authors:** A. S. Medeiros, A. Williams, D. Milošević

**Affiliations:** ^1^ School for Resource and Environmental Studies Faculty of Management Dalhousie University Halifax Canada; ^2^ Department of Biology Faculty of Science Trent University Peterborough Canada; ^3^ Department of Biology and Ecology Faculty of Sciences and Mathematics University of Niš Niš Serbia

**Keywords:** Arctic streams, benthic invertebrates, biological impairment, biomonitoring, Chironomidae, freshwater ecology, northern development

## Abstract

As increased growth and development put pressure on freshwater systems in Arctic environments, there is a need to maintain a meaningful and feasible framework for monitoring water quality. A useful tool for monitoring the ecological health of aquatic systems is by means of the analysis and inferences made from benthic invertebrates in a biomonitoring approach. Biomonitoring of rivers and streams within the Arctic has been under‐represented in research efforts. Here, we investigate an approach for monitoring biological impairment in Arctic streams from anthropogenic land use at two streams with different exposure to urban development in Iqaluit, Nunavut, Arctic Canada. Sites upstream of development, at midpoint locations, and at the mouth of each waterbody were sampled during 6 campaigns (2008, 2009, 2014, 2015, 2018, and 2019) to address spatial and temporal variability of the macroinvertebrate community. The influence of taxonomic resolution scaling was also examined in order to understand the sensitivity of macroinvertebrates as indicators in Arctic aquatic systems. We demonstrate that standard biological metrics were effective in indicating biological impairment downstream of sources of point‐source pollutants. A mixed‐design ANOVA for repeated measures also found strong interannual variability; however, we did not detect intra‐annual variation from seasonal factors. When examining metrics at the highest taxonomic resolution possible, the sensitivity of metrics increased. Likewise, when trait‐based metrics (α functional diversity) were applied to indicators identified at high taxonomic resolution, a significant difference was found between reference and impacted sites. Our results show that even though Arctic systems have lower diversity and constrained life‐history characteristics compared to temperate ecosystems, biomonitoring is not only possible, but also equally effective in detecting trends from anthropogenic activities. Thus, biomonitoring approaches in Arctic environments are likely a useful means for providing rapid and cost‐effective means of assessing future environmental impact.

## Graphical abstract

As increased growth and development put pressure on freshwater systems in Arctic communities, there is a need to maintain a meaningful and feasible framework for monitoring water quality. A useful tool for monitoring the ecological health of aquatic systems is by means of the analysis and inferences made from benthic invertebrates in a biomonitoring approach. Our results show that even though Arctic systems have lower diversity and constrained life‐history characteristics compared to temperate ecosystems, biomonitoring is not only possible, but also equally effective in detecting trends from anthropogenic activities.

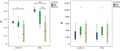

## 1 INTRODUCTION

Arctic freshwater systems are especially sensitive to both direct and indirect environmental stressors that can influence the trajectory of aquatic biota over time. As such, monitoring of Arctic ecosystems is difficult (Culp et al., 2012; Heino et al., 2020). Indirect long‐range transportation of contaminants, and the overarching influence of global climate, can be difficult to elucidate when direct point‐source influences are also a factor (Wrona et al., 2006). Seasonality is often considered the main driver of ecosystem dynamics within the Arctic—abrupt changes in solar radiation and temperature affect precipitation, snow, and ice conditions: all influencing the dynamic relationships which biota have with their environment (Medeiros et al., 2011; Prowse et al., 2006). Although broad trends in Arctic climate are important to consider, environmental monitoring programs inherently benefit from regional and locally based climate and habitat information to best suit their research objectives (Medeiros et al., 2011). Likewise, the inference of causal effects of impairment in Arctic streams requires consideration of the unique localized context, as local landscape characteristics are likely to cause changes in species richness, abundance, range, and distribution (Brittain et al., 2021; Vander Laan et al., 2013; Wrona et al., 2006).

The use of aquatic biota for monitoring aquatic ecosystems has led to the development of routine bioassessment programs that have been adopted by governance systems of many countries globally (e.g., RBP—Barbour et al., 1999; AQEM/STAR—AQEM, 2002; Buss et al., 2015). Heino et al. (2020) note the importance of a standard approach to bioassessment for Arctic freshwater ecosystems; however, there are very few studies that have successfully used standard approaches in Arctic North America. Models that link biological indicators to a degradation gradient often have a high level of unexplained variability, also called “noise,” which can be interpreted as a natural variability (Cid et al., 2020; Milošević et al., 2013). Biological metrics could also vary strongly along spatial and temporal scales, which can create difficulties in disentangling natural from man‐caused variability (Milošević et al., 2013; Soria et al., 2020). For example, species dispersal ability is known to be a significant factor in determining the response to seasonal factors that affect flow intermittence in small streams (Cid et al., 2020). Yet, Kärnä et al. (2015) found that environmental differences correlated to the dissimilarity of insects more than dispersal limitations from physical distances across subarctic streams in northern Finland and Norway.

The level of taxonomic identification necessary for bioassessment depends on the specific objectives of the research question and could also potentially influence conclusions of biological assessment, especially in highly heterogeneous systems (Trigal‐Domínguez et al., 2010). Species‐level identifications will offer a robust dataset with a large amount of scientific information, yet this may not be feasible; fine‐scale taxonomic identification requires a large amount of specialized training and is associated with a high cost (Jones, 2008). Conversely, genus‐ or family‐level identification can be used if it is deemed sufficient in terms of information gained (Jones, 2008). For Arctic environments, where abundances and diversity are known to be low, increased taxonomic resolution may be necessary to detect fundamental shifts in biological composition related to the environment (Medeiros et al., 2011). Likewise, high taxonomic resolution of indicators can expand the depth of understanding the extent of impacts, as additional trait‐based metrics can be used to infer structural changes in functional niche (Mason et al., 2005). For example, for Arctic environments, functional redundancy is expected to be high due to environmental limitations on dispersal (Brown et al., 2018); yet, impairment is expected to result in functional homogenization in streams (Voß & Schäfer, 2017). Here lies an opportunity, increased taxonomic resolution and trait‐based metrics of biological indicators in Arctic systems could provide a means for understanding the influence of both direct point‐sources of impairment, but also provide a means for biomonitoring to be sensitive enough for understanding the greater context of climate change (Gallagher et al., 2013; Mayfield et al., 2020).

While aquatic monitoring programs that focus on benthic macroinvertebrate communities are standard for inclusion in the assessment of freshwater ecological integrity (Hering et al., 2006), policy and governance surrounding Arctic freshwater resources are fragmented and, in some cases, nonexistent (Medeiros et al., 2017). Heino et al. (2020) note the urgent need for monitoring Arctic freshwater ecosystems, especially with respect to the unknown and often unpredictable influence of environmental change, where the data produced by baseline studies are integrated into informed and realistic planning processes at both local and regional scales of governance. A lack of continuous planning and enforcement undermines the intent of such programs and devalues community input (Noble & Hanna, 2015). Public perception and awareness fuel community discourse surrounding freshwater; justified concerns pertaining to water quality have instilled relatively new interest in monitoring the sustainability of these complex systems (Bakaic et al., 2018).

Here, we address land‐use impacts on aquatic biota in streams of Iqaluit, Nunavut, Arctic Canada. We examine both temporal variability and spatial differences along a gradient of anthropogenic disturbance as streams pass through urbanized areas. Taxonomic sufficiency is also considered to test the variability of benthic macroinvertebrate assemblages as indicators of ecosystem degradation. While there is a lack of existing baseline data and knowledge of lotic ecosystems in the Arctic, the use of studies that compare impacted sites against control and reference sites are known to be effective in this context (Medeiros et al., 2011). As such, in order to quantify biological impairment of northern streams, we (1) examine temporal and spatial variation in macroinvertebrate assemblages, (2) determine the variability of macroinvertebrates via diversity indices and trait‐based metrics along a degradation gradient, and (3) determine the influence of taxonomic resolution on the sensitivity of macroinvertebrates as a reliable indicator of impairment for Arctic streams. We examine two streams, one of which transitions through a large, well‐known, urban disturbance gradient, and another that has had increased influence from urban development along its reach in the past decade, but no evidence of direct point‐source impacts.

## 2 METHODS

### 2.1 Study area

**FIGURE 1:**
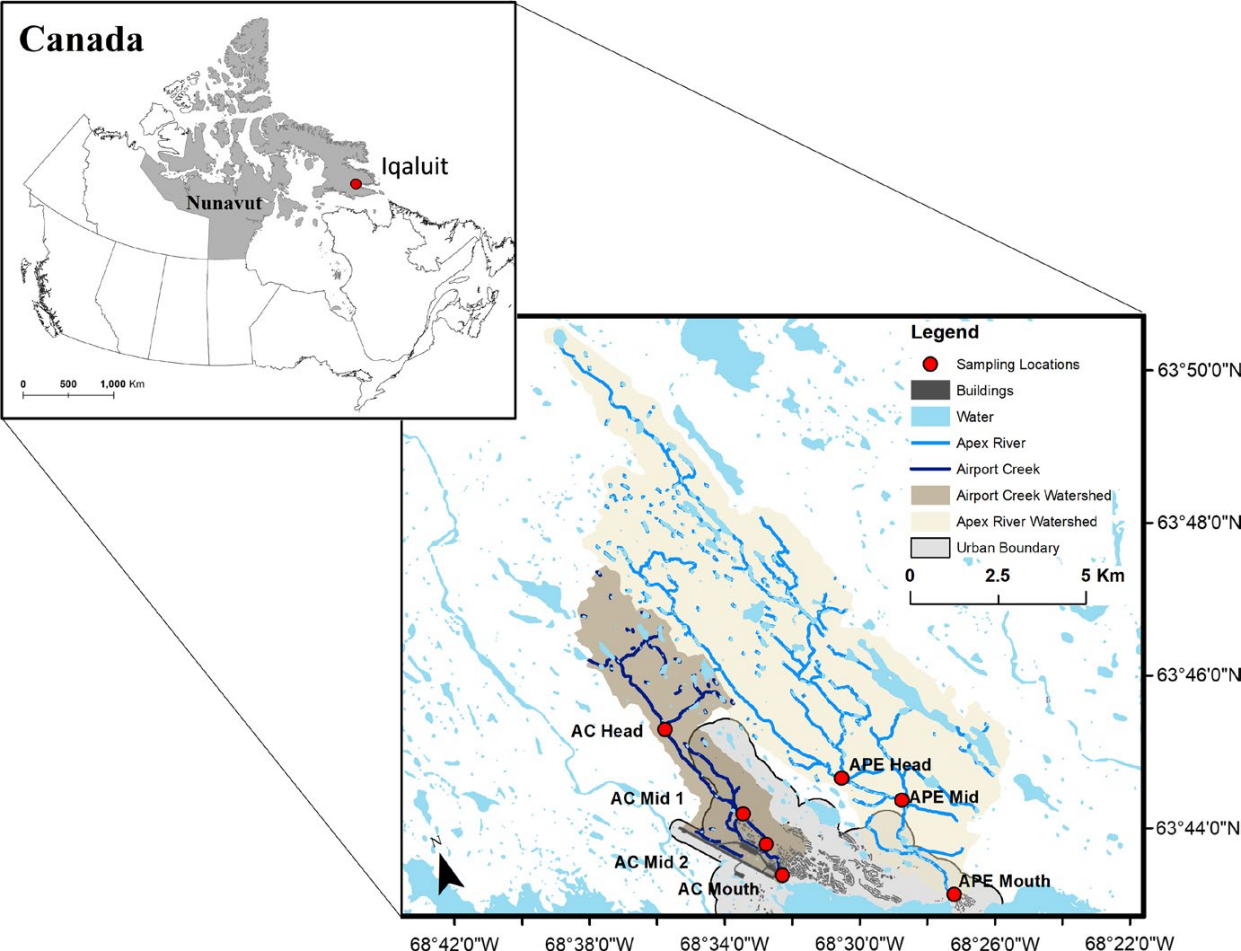
Location of sampling sites for the Apex River (APE) and Airport Creek (AC) in Iqaluit, Nunavut. The delineation of the Apex River Watershed (tan) and Airport Creek Watershed (brown) is shown to highlight the overlap between the watershed, sampling locations, and urban boundary. Data sources are described in supplemental information

The City of Iqaluit, NU (63.7467 °N, 68.517 °W), is located in Nunavut, Arctic Canada (Figure 1). The region is within the continuous permafrost zone and characterized by a high degree of elevation change with numerous streams, shallow ponds, and lakes (Medeiros et al., 2011). Two streams, the Airport Creek and Apex River (Figure 1), were assessed through a biomonitoring study that began in 2007 as a community‐based monitoring program (Medeiros et al., 2011). The study was designed to assess impacts of differing land‐use and development stressors such as point‐source pollutants in an effort to quantify biological impairment and compare the impacted stream to the control site. Measures of water quality and the associated classification of sites are described in Medeiros et al. (2011). Since 2007, Iqaluit has grown by ~1,600 residents and expanded its urban influence toward the Apex River; no remediation efforts have occurred.

Airport Creek (known locally as Carney Creek) is a 3^rd^ order stream that is surrounded by multiple land‐use stressors within the downstream portion of the creek and drains a total area of about 16.5 km^2^. The stream has negligible flow during drier periods and freezes to the bottom in winter months. The substrate is glacially derived alluvial deposits in the headwater areas that become increasingly dominated by gravel and silt in the urbanized zones. Within the urban zone, the river has been crudely channelized in areas and contains several culverts and overpasses from local gravel roads. The headwaters are currently outside of the zone of human influence but are being increasingly encroached upon by development as land‐use extends toward the headwaters of the catchment area (Figure 1). The Apex River is a 4^th^ order river that flows east of the City of Iqaluit. The river continuously flows throughout the year, including large portions that flow beneath the ice in the winter months. The substrate is glacially derived alluvial deposits in the headwater zone that become influenced by increasing amounts of silt and clays as the river braids at its midpoint. The river catchment drains a comparatively larger watershed of 60 km^2^. Anthropogenic impacts are minimal comparatively, but include two gravel pit sites located along the east bank of the river, an active gun range, an expanding residential development, and several areas that are used as local campgrounds. The habitat surrounding both rivers is similar in their headwater areas, comprised of typical tundra conditions. See supplemental information for a further description of the study area.

### 2.2 Field and laboratory methodology

Benthic macroinvertebrates were sampled at three locations: headwaters (upstream of urban zone), midpoint (the center‐point of urban influence), and mouth (outlet, above tidal zone influence)—along Airport Creek and Apex River in the summers of 2008, 2009, 2014, 2015, 2018, and 2019. We note that for 2008, 2009, and 2014–2015, the midpoint location was sampled twice along two slightly different areas along reach of Airport Creek (Figure 1), which was later amalgamated into a single midpoint sample in 2018 and 2019. For ease and consistency of our analysis and discussion, we have amalgamated the two sampling locations into a single midpoint composite sample. While the rivers were sampled in 2007 and 2010–2013, the sampling effort was not sufficient to allow for a high‐resolution taxonomic examination of indicators. The years 2016, 2017, and 2020 represent gaps in the community‐based monitoring program. At each location, invertebrates were collected from three transects—a riffle, pool, and a run. Samples were collected using methods for Arctic waterbodies outlined in Medeiros et al. (2011), including a 5‐min kick‐and‐sweep survey using a 425‐µm D‐net. Samples were carefully washed out of the D‐net into a wash bin and transferred into previously washed polyethylene bottles. Samples were taken in cycles—shortly after the seasonal melt, mid‐summer, and late‐summer, but sampling periods were not consistent year‐year due to seasonal variability and logistical constraints (Table S1).

Aquatic insect taxa were sorted and enumerated at the Nunavut Research Institute in Iqaluit with the aid of a stereomicroscope to ensure smaller taxa were found within thick sands and macrophytes. Sorted specimens were then preserved in 95% ethanol and transported to Dalhousie University for further analysis. For all years, macroinvertebrates were re‐counted and identified to the family and subfamily/tribe level of taxonomic resolution (following Medeiros et al., 2011), except in 2019 where specimens were identified up to the genus, subgenus, or species level using Oliver and Roussel (1983), Merritt et al. (2008), and Andersen et al. (2013). For all samples, and all collection periods, the complete 5‐min sample was enumerated; no subsampling methods were conducted.

### 2.3 Data analysis

In order to qualify potential impairment in each stream, we created three data matrices from enumerated macroinvertebrates for a comparison of trends (Table S1): a spatial–temporal matrix, a seasonal–temporal matrix, and a taxonomic scale (taxonomic sufficiency) matrix. For all types of matrices, we calculated the following diversity indices as a biotic metric: species richness (S), abundance (N), Shannon index (H), and Pielou evenness index (J′).

The spatial–temporal matrix comprised six campaigns, with multiple monthly surveys (June, July, and August, where applicable), where a single campaign encompassed all sampling periods conducted in a year. Macroinvertebrates were compared at the family/subfamily level to determine whether the macroinvertebrate community via diversity‐based metrics (taxonomic richness, abundance, and diversity indices) changed along spatial or temporal gradients. A two‐way repeated measures test and mixed model analysis of variance (mixed‐design ANOVA) with post hoc Bonferroni correction were conducted on six campaigns where samples were collected during July of each year (i.e., 18 taxa x78 samples) in SPSS 19 (Norusis, 2012). Models were generated with the temporal (different years of the campaign) and spatial (different river sections) gradients as a within‐ and between‐subject, respectively.

The spatial–temporal matrix was also used to examine the variability of macroinvertebrates across the degradation gradient, where diversity‐based metrics (taxonomic richness, abundance, and diversity indices) were compared with a one‐way ANOVA and Kruskal–Wallis test applied on each campaign individually, including multiple monthly surveys (June, July, and August, where applicable, 18 taxa × 144 samples). For pairwise comparisons of sampling sites, Mann–Whitney and Fisher's least significant difference (LSD) post hoc tests were used. The same matrix was also used to analyze the differences in community structure between sampling locations; enumerated specimens were compared in a two‐way crossed distance‐based permutational multivariate analysis of variance (PERMANOVA; Clarke & Warwick, 2001) with a Bray–Curtis resemblance matrix using the Primer statistical package (v61.14), see supplemental information for a more detailed description of these analyses. The variability pattern of community structure was visualized via principal coordinate analysis (PCoA) plots.

The seasonal temporal matrix encompassed the 2018 campaign with the highest number of sampling occasions in the study. Samples were evenly distributed in all three months (June, July, and August, 18 taxa x47 samples). A one‐way ANOVA was used for comparison between sampling periods. The abundance of each taxon was log‐transformed, while all other input parameters fulfilled the assumptions of normality, sphericity, and homogeneity.

The third matrix examined fine (genus, subgenus, and species level; 66 taxa) and coarse (family/subfamily level; 18 taxa) taxonomic resolution of macroinvertebrates in 35 samples from the 2019 campaign. The influence of taxonomic resolution on the sensitivity of macroinvertebrates was tested using a taxonomic matrix, where differences in diversity‐based metrics and community structure between sampling sites in 2019 were determined by one‐way ANOVA and Kruskal–Wallis, and one‐way PERMANOVA, respectively. The differences in community structure obtained by PERMANOVA were then visualized by PCoA.

Using the fine‐resolution identification of taxonomy from the 2019 campaign, we then compared samples using functional diversity. Functional diversity is presented as α functional diversity (FD), functional evenness (Feve), and functional dispersion (Fdis) calculated using the BAT package (Mammola & Cardoso, 2020). The variability of functional diversity was analyzed in functional n‐dimensional hypervolumes, which were constructed for each sample. Functional hypervolumes were obtained through the *hypervolume* package (Blonder, 2019). A trait matrix was constructed assigning each taxon to their associated functional feeding group (FFG), biological group, and ecological group (Table S2). The FFG presents feeding behavior and are assigned to one of 5 categories: gatherers/collector (Cg), active filter feeder (F), predators (P), scraper (Sc), and shredders (Sh); as outlined in AQEM (2002), Buffagni et al. (2021), Graf et al. (2021), and Brabec et al. (2021). Seven biological groups were used to describe the life‐history traits (size, life‐cycle, respiration, reproduction, and locomotion; Usseglio‐Polatera et al., 2000). The ecological groups were outlined where indicators are associated with ecological requirements (distribution, favorable substrate, and current velocity; Usseglio‐Polatera et al., 2000). The Gower dissimilarity measure was used to convert trait‐based metrics into a distance matrix, which was used to extract orthogonal morphological axes. The first three principal coordinate axes are used to construct hypervolumes using a Gaussian kernel estimator (Blonder et al., 2018). Alpha functional diversity was calculated using the function *kernel.alpha,* which presents the total volume of the functional hyperspace, filled by species in the community (Mammola & Cardoso, 2020). Functional evenness was calculated by the function *kernel.evenness* and represents the regularity of the distribution of functional elements within the total trait space (Mammola & Cardoso, 2020; Mason et al., 2005). Functional dispersion was calculated using the function *kernel.dispersion* to obtain the average differences between the trait space centroid and random points within the hypervolume. Functional dispersion is an analog for functional divergence, which determines the distribution of species abundance in niche space and maximizes divergence in functional elements of the community (Mason et al., 2005). Differences in functional diversity‐based metrics between the two rivers were tested with a Mann–Whitney test.

## 3 RESULTS

### 3.1 Natural variability of benthic macroinvertebrates across the temporal scale

**FIGURE 2:**
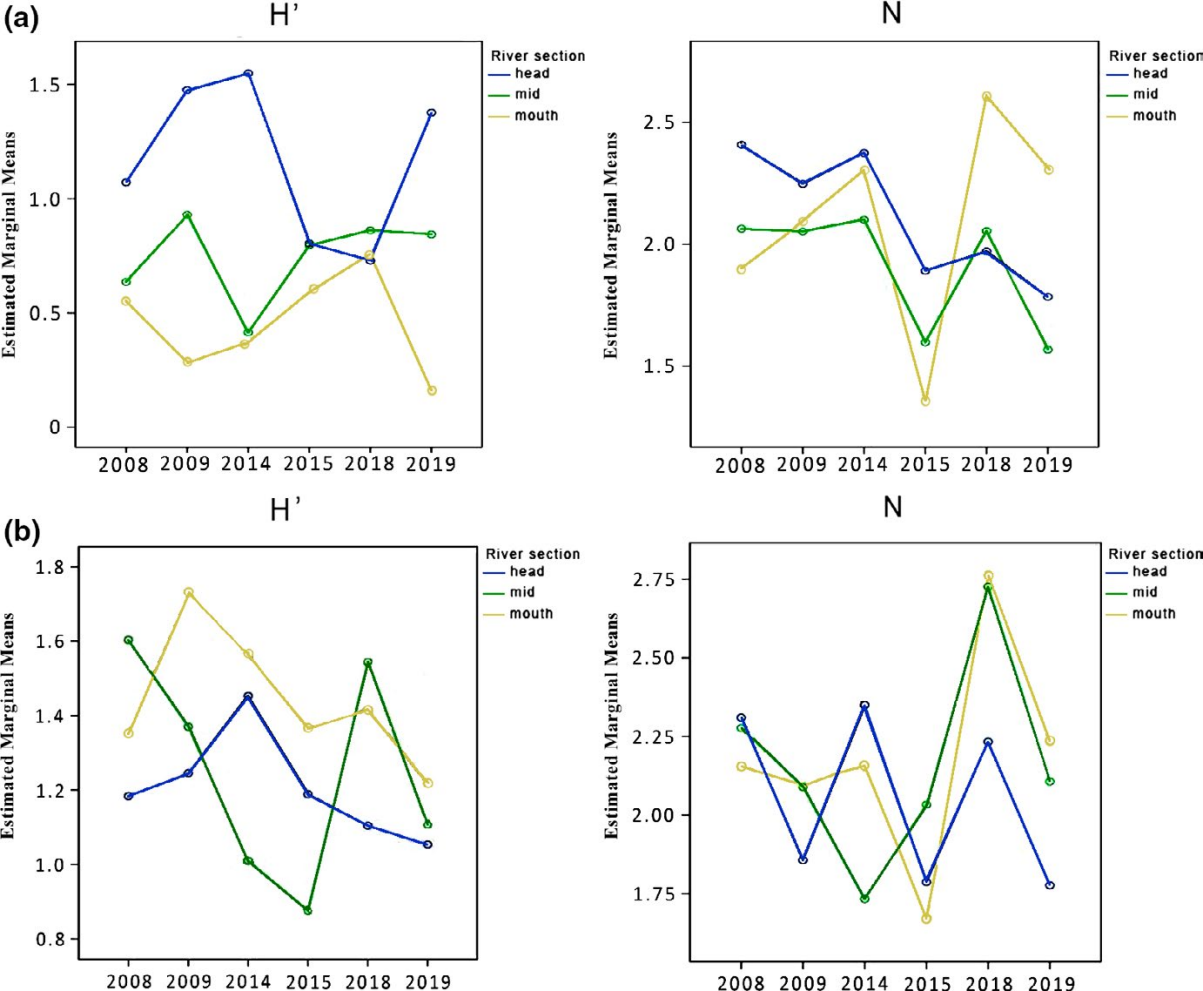
Interaction between within‐subject (time) and between‐subject (river section) factors, presenting mean scores of diversity indices (Shannon index (H′) and abundance (N), for (a) Airport Creek and (b) Apex River

Species richness (S) in Airport Creek changed significantly between river sections (mixed‐design ANOVA for repeated measures, *F* = 12.094, *p* = .008), while the abundance of invertebrates showed significant variability only across temporal scales (*F* = 5.761, *p* = .001). However, both species richness and abundance had significant interactions between time (years) and sampling location (for S, *F* = 3.780, *p* = .002; for abundance, *F* = 2.315, *p* = .037). A post hoc pairwise comparison found that species richness significantly differed between river sections during 2008, 2009, and 2014 (*p* = .0001). The largest difference between sampling locations occurring in 2009, where 10, 7.6, and 3.3 species (on average) were recorded on headwaters, mid, and mouth sections, respectively (Figure S1a). In 2008, the midpoint and mouth of the river had a significantly lower number of species (an average of 3.6 on both sections) than the headwaters site (7.6), while in 2014 only headwater and midpoint locations significantly differed (7.3 and 4.6, respectively; *p* = .004). The variability of species richness became monotonous during 2015 and 2018 and increased again in 2019 where the headwater (7) and midpoint (7.6) sections had a similar but significantly higher number of species than the mouth section (3.3; *p* = .004). Between 2009 and 2014, species richness of the headwater and midpoint locations significantly decreased from an average of 10 and 7.6 to 7.3 and 4.6 taxa, respectively (*p* = .012 and *p* = .006), while conversely increasing at the mouth location from an average of 3.3 to 6 species (*p* = .012; Figure S1a). The abundances of invertebrates at the mouth of the river varied as a consequence of the interaction between time and position (2014–2015, *p* = .011; 2015–2018, *p* = .003; Figure 2a).

The diversity of macroinvertebrates of Airport Creek varied significantly between river sections (for H’, *F* = 76.880, *p* < .001; J’, *F* = 12.167, *p* = .008). When averaged across sampling sites, diversity was not significantly different between sampling years; however, we found a significant interaction between factors, time (years), and group (river section), for all diversity indices (for H’, *F* = 3.945, *p* = .002; J’, *F* = 3.100, *p* = .008). In 2009, diversity (H’) significantly decreased downstream (4.42, 2.78, and 0.93 from the headwater site to the mid and mouth sections, respectively; post hoc Bonferroni, *p* < .001). However, these differences were less obvious in 2008 and 2014, where only the headwater sites were higher (1.07 and 1.54) than mid (0.63 and 0.41) and mouth locations 0.55 and 0.37, respectively (*p* < .01). These differences were not apparent in 2015 or 2018 as the diversity of the headwater location significantly decreased (*p* < .05) from 1.54 in 2014 to 0.80 in 2015 and 0.72 in 2018 (Figure 2a). The diversity of the headwater section increased again to 1.37 in 2019, where only the mouth section had significantly lower diversity (0.15; *p* < .01). The Pielou evenness index showed a similar pattern wherein 2009 all sections significantly differed (0.64, 0.45, and 0.23 for headwaters, mid, and mouth sections, respectively: *p* < .001). However, by 2014, the mid and mouth sections had similar lower values (0.27 and 0.20), which were also lower (*p* < .001) than the headwater sampling site (0.77; Figure S1a). By 2019, only mouth of Airport Creek had significantly lower (*p* < .01) values (0.13). A one‐way ANOVA revealed the absence of seasonality showing nonsignificant variation among all tested matrices (S, logN, H′, and J′, *p* > .05) between sampled months (Jun, July, and August).

For the Apex River, diversity was not found to differ significantly over the time or between sampling locations (*p* > .05). However, the abundance of invertebrates was found to vary across time (mixed‐design ANOVA, *F* = 4.405, *p* = .004), increasing between the two sampling campaigns in 2015 and 2018 (post hoc Bonferroni, *p* < .05; Figure 2b). Similar to Airport Creek, there was no significant variation detected between sampled months (Jun, July, and August).

### 3.2 The variability of macroinvertebrates along the degradation gradient

**FIGURE 3:**
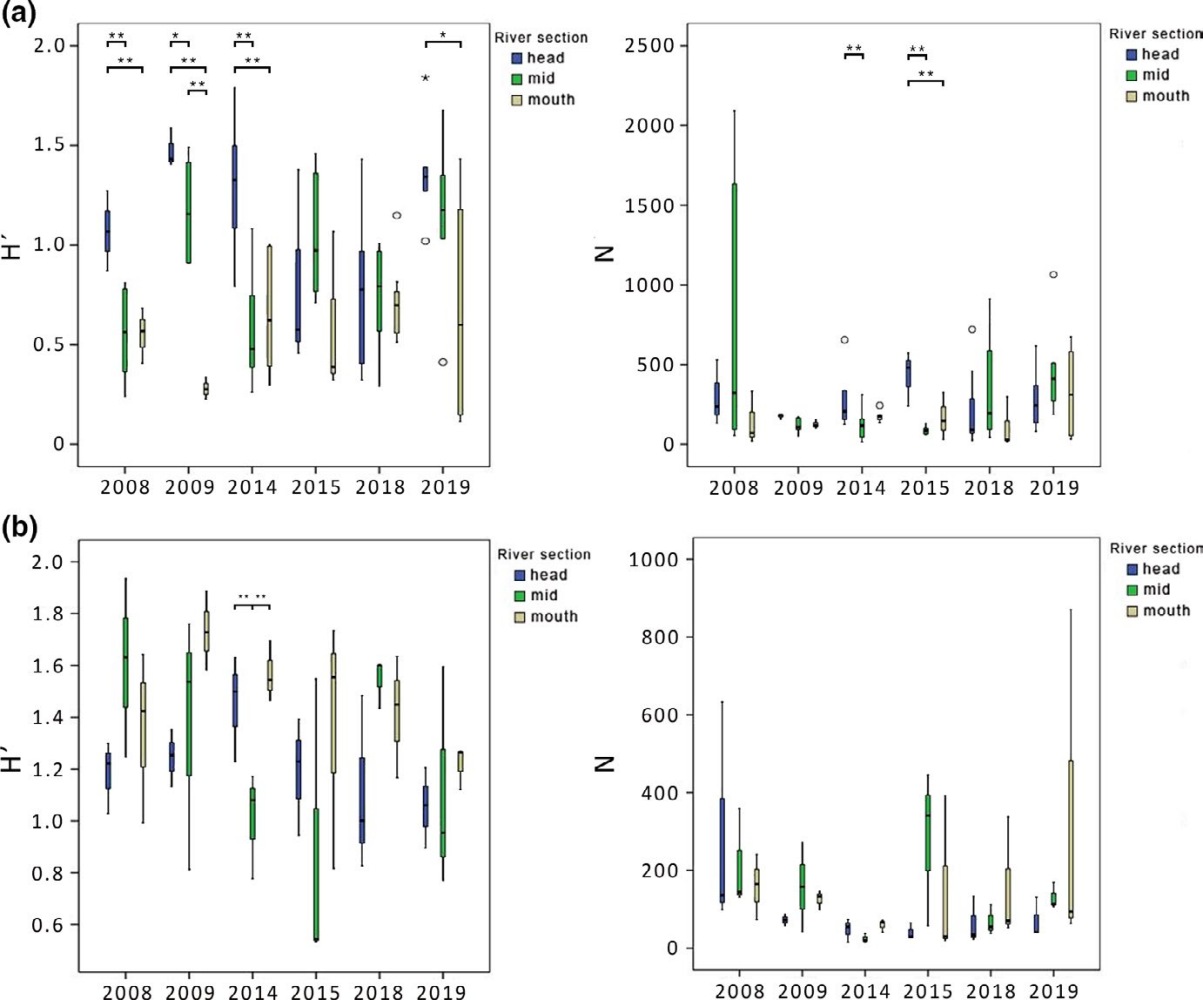
Boxplot representation of diversity indices ((Shannon index (H′) and abundance (N)), across rivers section (head, mid, and mouth) and time (2014, 2015, 2018, and 2019) of (a) Airport Creek and (b) Apex River. Significant differences are noted (*p < .05, **p < .01)

When comparing samples collected during different periods of the same year (June, July, and August) for 2008–2019, species richness was found to vary significantly across each sampling location for Airport creek for all years except 2015, while abundances were found to be significantly different between river sections in only 2014 and 2015 (Kruskal–Wallis, *p* < .05; Figure 3a; Figure S2a). When measures of diversity were compared, a one‐way ANOVA indicated a similar result as the mixed‐design ANOVA (Figure 2). Differences in diversity indices between river sections were found for 2009 (for H’, *F* = 27.381, *p* < .001; J′ and *F* = 7.026, *p* = .015) and 2014 (for H’, *F* = 13.284, *p* < .001 and J’, *F* = 6.506, *p* < .006), while in 2008 and 2019 only the Shannon index varied significantly (*F* = 6.889, *p* = .015 and *F* = 3.717, *p* < .05, respectively). A decreasing trend for diversity along Airport Creek was present in all years but 2015 and 2018, where sampling sites had a similar level of diversity (*p* > .05; Figure 3a and Figure S2a).

A distance‐based permutational multivariate analysis of variance (PERMANOVA) found that changes in community structure across sampling sites (pseudo‐*F* = 8.494, *p* < .001) and years (pseudo‐*F* = 4.244, *p* < .001) were significant. The first two axes of a PCoA explained 42.1% of the variance of community structure along spatial and temporal scales as determined by PERMANOVA (see Figure S3). The most pronounced differences were observed in 2009, 2014, and 2019, where sampling sites showed a clear tendency of grouping according to sampling location. For samples collected in 2008, 2015, and 2018, sites overlapped within the ordination space, indicating little variation between samples.

When sampling campaigns were compared for the Apex River, diversity only varied significantly in 2014 (one‐way ANOVA, *F* = 7.943, *p* = .021). This difference was only observed at the midpoint sampling location, where diversity was significantly lower than the headwaters or mouth of the river (Figure 3b). Evenness of macroinvertebrates from the headwater sampling location was lower in 2008 (*F* = 6.385, *p* = .033; Figure 5). While differences in the macroinvertebrate community were found across time and river sections (PERMANOVA, pseudo‐*F* = 8.494, *p* < .001; pseudo‐*F* = 8.494, *p* < .001, respectively), PCoA revealed that there was no clear tendency of grouping of any river section or sampling year (see supplemental Figure S4).

**FIGURE 4:**
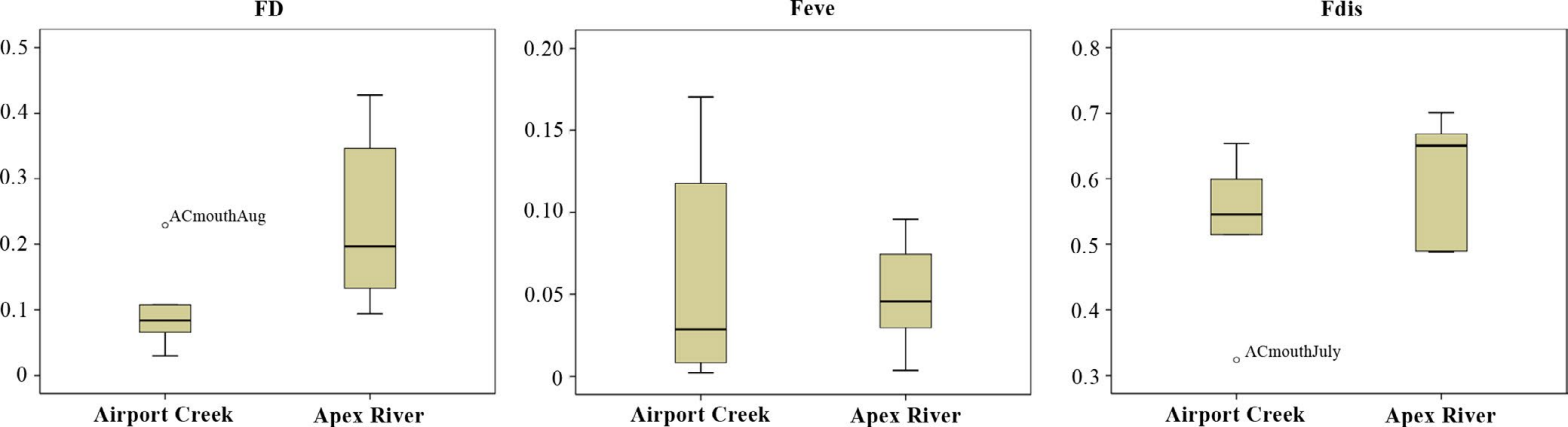
Boxplot of functional diversity indices comparing α functional diversity (FD), functional evenness (Feve), and functional dispersion (Fdis) for invertebrates collected in 2019 from the Apex River and Airport Creek, Iqaluit, Nunavut

Of the three functional diversity metrics examined, only α functional diversity (FD) significantly varied between two rivers (Mann–Whitney test, *p* = .041). FD was higher in the Apex River (0.23 ± 0.12) than in Airport Creek (0.10 ± 0.06). Functional evenness and functional divergence were similar for both rivers (Figure 4).

### 3.3 The sensitivity of bioassessment metrics at different taxonomic resolution

To determine the influence of taxonomic resolution on the sensitivity of diversity indices as a metric for bioassessment, collected specimens from 2019 were identified to the highest taxonomic resolution possible resolution possible (Table S2). A PERMANOVA comparison of the differences in community structure across the river sections found significant differences for assemblages identified at both taxonomic levels: coarse (pseudo‐*F* = 6.323, *p* < .001) and fine resolution (pseudo‐*F* = 4.832, *p* < .001). While differences were significant for both taxonomic levels, a PCoA revealed that the community structure described with fine taxonomic resolution ordinated and grouped sampling sites more distinctly, separating all three sections of the river (see Figure S5).

**FIGURE 5:**
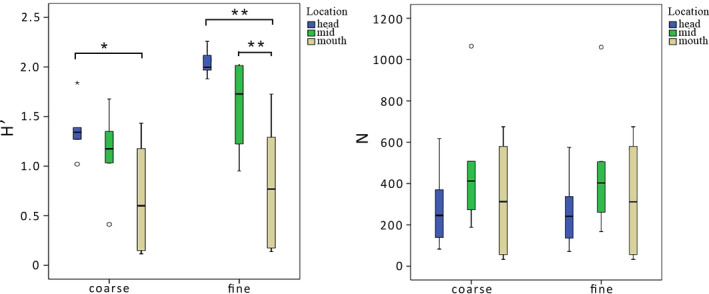
Boxplot of diversity indices: Shannon index (H′) and abundance (N), based on the data sets with coarse (family/subfamily‐level) and fine (species‐level) taxonomic resolution for Airport Creek. LSD and Mann–Whitney post hoc tests were applied for post hoc comparison (*p < .05, **p < .01)

Diversity was found to vary significantly between river sampling locations (for H, *F* = 9.855, *p* < .01; J’, *F* = 4.936, *p* < .05). In contrast, a post hoc pairwise comparison revealed that these differences were not always readily observed at a coarse taxonomic resolution (Figure 5 and Figure S6). For coarse resolution data, diversity differed only between the headwater (8.20, *p* < .05) and mouth sections (4.07, *p* < .05). Species richness (S) was understandably higher with an increased taxonomic resolution of identification, but the pattern of change was similar, where headwater and mid sections of the river contained a higher number of species (average of 6.8 and 7.5 taxa for course resolution and 14.8 and 15.16 taxa for fine resolution), and the mouth of the river contained less (4.5 taxa for course resolution and 8.1 taxa for fine resolution; Kruskal–Wallis, *p* < .01).

## 4 DISCUSSION

As the diversity in Arctic streams is inherently low compared to temperate areas, where biomonitoring programs are able focus on rapid family‐level identification of the benthic community, less information for distinguishing sites is available (Medeiros et al., 2011). While our results are similar to the initial study by Medeiros et al. (2011), we further examined assessment criteria, metrics, and inferences that qualify biological impairment in Arctic streams by (1) testing the variability of macroinvertebrates presented via diversity indices and community structure along a spatial gradient, (2) testing the extent of natural variability that may affect macroinvertebrate communities along a temporal gradient, and (3) determining the influence of taxonomic resolution on the sensitivity of our interpretations.

### 4.1 Variability along space

Assessing stream health can be a challenge due to the presence of multiple stressors from an array of natural and anthropogenic sources. Depending on the spatial extent (the size of the region encompassing all localities in a region unit; Heino et al., 2015), the natural variability of aquatic macroinvertebrates encompasses environmental drivers, dispersal processes, and biotic interactions that can all influence community structure differently. This is especially true where natural variation in either biological indicators or environmental conditions is high (Mousavi et al., 2003; Poulton et al., 1995), such as in Arctic environments (Medeiros et al., 2011). We confirmed significant anthropogenic‐related impairment of Airport Creek as was identified by Medeiros et al. (2011); the pattern of impairment through the urbanized portion of Iqaluit was present in all years and in all biomonitoring metrics tested. However, natural variability in space was difficult to ascertain considering the scale of the present study (only two rivers included) and the length of environmental gradients. According to AQEM protocols (a standard assessment model in European waters), an ideal metric should be responsive to stressors with low natural variability, which would have negligible confounding effects from an anthropogenic‐caused degradation gradient (Hering et al., 2006). Vinke et al. (2015) also noted large seasonal variation was an important determinant of benthic macroinvertebrate assemblages for streams in subarctic Canada, but were still able to qualify habitat‐level characteristics associated with a high‐resolution examination of indicators.

All bioassessment programs worldwide are based on a niche‐based approach, where metrics are solely controlled by local environmental factors (Heino, 2013; Vilmi et al., 2016). However, this is supported by the fact that previous studies, especially for lotic systems, showed that environmental controls are the main force in structuring aquatic communities, including macroinvertebrates (Heino et al., 2017). We note that the Apex River, for the most part, did not show signs of impairment from anthropogenic activities along its reach and also did not show any changes that could be considered due to natural variation along its spatial extent. The premise for bioassessment is to describe the covariance of degradational gradients with the natural ones, which can hinder precise indication of anthropogenic pressures by biological components of aquatic ecosystems (Pereira et al., 2016; Ruaro et al., 2020). However, the absence of natural spatial variability found in the context of the Apex River does not mean that it does not exist in Arctic streams, but could be both an artifact of the sampling design applied here as well as a larger influence of regional effects that cascade through all points of these small nival systems (Docherty et al., 2018). Therefore, spatial processes and biological interactions may be considerations in studies with a larger spatial sample (Cid et al., 2020), or watersheds covering large and heterogeneous regions with larger gradients of local or other factors that may enhance natural variability (Miller et al., 2016; Stoddard et al., 2008).

### 4.2 Variability over time

Interannual and seasonal variability, as well as the timing of the sampling period, plays a large role in determining the abundance of benthic invertebrates collected using biomonitoring approaches (Hawkins et al., 2010). Depending on yearly precipitation and heat, the abundance of assemblages can vary greatly since there is a clear relationship between variability of macroinvertebrate α diversity and air temperature variation (Culp et al., 2019); this is especially true in Arctic environments where seasonality is known to have significant control over invertebrate abundances (Danks, 2007). Indeed, for Arctic environments, there is an expectation that the natural temporal variability of invertebrates is high (Meyer et al., 2017) and could be based on stochastic factors (Miller & Stout, 1989). Høye et al. (2021) also note that recent decreases in diversity and increases in abundance are likely linked to environmental change; yet, the overall trends may not necessarily be linear through time.

Interannual fluctuations in diversity metrics were noticeable in our study. A significant decrease in the number of species was recorded in 2014 while the same trend was observed for diversity and abundance in 2015 and 2018 (Figure 2 and Figure S1). This contrasts with the expected increase in species richness as a consequence of regional warming (Lenato et al., 2019). We recorded diversity in both sampled rivers that fluctuated through time and had no discernable association with climate. The lack of an interannual pattern is likely a consequence of factors associated with different scales: atmospheric processes (such as climate), regional factors (such as regional pollution or catchment characteristics), and local factors (Gaston et al., 2000). For example, the large influence of a local greenhouse that was shown to be discharging phosphorous enriched water to Airport Creek was decommissioned in 2014, which is likely a reason for lowered abundance of macroinvertebrates downstream of this site in the following years. Likewise, a perpetual garbage fire that burned for much of 2014 (referred to locally as dumpcano) had a significant effect on regional air for Iqaluit (Weichenthal et al., 2015), which would have affected both streams equally across all points. We should also note that our study focused on a coarse taxonomic resolution (family‐level) comparison of annual trends, which does not often have the ability to differentiate interannual variation. The species–environment relationship may also strongly differ between species of the same genera in species‐rich families, such as is known for chironomids (Medeiros & Quinlan, 2011; Milošević et al., 2013).

Intra‐annual seasonal variation of aquatic macroinvertebrates is a consequence of large life‐history differences among the community's constituent taxa and can strongly influence temporal variation (Johnson et al., 2012). This is especially true for chironomids (Milošević et al., 2013), which are the dominant benthic invertebrates in Arctic aquatic ecosystems (Danks, 1992). Differences in species richness of chironomids are known to be variable on seasonal scales as emergence patterns are different between different subfamilies belonging to either spring or summer forms (García & Suárez, 2007; Milošević et al., 2013). Indeed, temporal variability of chironomids could be so strong that year‐to‐year differences in relative abundance of chironomids can exceed site‐to‐site differences (Kerans & Karr, 1994). Despite this, our results show an absence of intra‐annual seasonal variability. For Arctic streams, this is not entirely surprising as thermodynamic processes constrain phenological processes (Danks, 2007). For most Arctic aquatic insects, rapid development in summer and a long dormancy in other periods is a life‐history strategy for survival in extreme climates (Danks, 2007). This results in an extended duration of macroinvertebrate life‐cycle stages and a single generation per year (univoltine species). In addition, many Arctic species avoid synchronized development of populations, exhibiting scattered emergence (Ulfstrand, 1969). Such a unified phenology pattern as well as a more gradual and asynchronized emergence likely decreases seasonal variability of indicators (during the ice‐free period).

### 4.3 Taxonomic factors

All routine biomonitoring programs are built upon a trade‐off between “as high as possible” taxonomic resolution in data and acceptable cost‐effectiveness of the method (Verdonschot, 2006). This is due to the time‐consuming nature of identification of macroinvertebrates to a higher taxonomic resolution (Jiang et al., 2013). However, low diversity in Arctic streams does suggest that species–environment relationships may ultimately be important enough to justify further examination of trends at high taxonomic resolution (Medeiros et al., 2011).

We identified 62 taxa when examining specimens to the highest possible taxonomic resolution in 2019. If we applied a course resolution (family‐level identification), there would only be 18. The difference in richness influenced the ability of standard biomonitoring metrics to discern differences between sampling location and periods (Figure 5 and Figure S6). In contrast, differences between samples were more pronounced when a high‐resolution approach was used to ordinate sampling locations, indicating a clear tendency of sample grouping (Figure S5). We note that from the 67 identified taxa in 2019, 54 belong to the Family Chironomidae (Table S2). For Arctic streams, this is the common dominant group; yet, the Chironomidae are also most frequently not identified beyond the family‐level resolution in biomonitoring approaches (Jones, 2008; Reynoldson & Metcalfe‐Smith, 1992). Milošević et al. (2020) note that obstacles in the identification of chironomids have resulted in their exclusion in routine bioassessment, which has resulted in Chironomidae referred to as a *dark taxon*. Likewise, Raunio et al. (2011) note that freshwater surveys may be biased without the inclusion of chironomid indicators, as they are known to be especially responsive to anthropogenic disturbances and can increase the signal‐to‐noise ratio for bioassessment.

Increased taxonomic resolution also allows for insights into trophic position and the associated functional traits that help determine how interactions occur in the environment. Indeed, functional diversity has been recognized as an important characteristic of biological communities (Mason et al., 2005). Biodiversity loss as a consequence of ecosystem deterioration can lead to structural changes in functional niche, eventually homogenizing trait composition within the community (Brown et al., 2018; Elias et al., 2015; Piano et al., 2020). The functional aspects of recovery could be used in biomonitoring by comparing predictable changes in functional diversity along environmental gradients as well as comparing co‐occurring groups (Berger et al., 2018; Cai et al., 2019; Menezes et al., 2010; Van den Brink et al., 2011). Trait‐based metrics are also more stable on the regional scale (Brown et al., 2018; Cai et al., 2019; Statzner & Beche, 2010; Van den Brink et al., 2011) and not influenced by biogeographical constraints, unlike the taxonomic responses (Medeiros et al., 2021).

We found a clear difference in functional diversity between the Apex River and Airport Creek, while functional evenness and functional dispersion were not found to be different. Unlike temperate regions, Arctic systems have naturally depauperate assemblages (Danks, 2007). Mayfield et al. (2020) found that colder freshwater ecosystems had lower beta diversity of chironomids indicators, suggesting reduced turnover and increased predictability between assemblages across spatial gradients. Ultimately, environmental stress in Arctic ecosystems also inherently leads to habitat homogenization and fewer available niches environmental stress, due to habitat homogenization and few available niches (Wang et al., 2019), which could potentially overshadow any differences realized from additional anthropogenic stress. That being said, monitoring differences in trait‐based metrics could become important under continued warming that increases in beta diversity leading to increased stability in ecosystems that are not under direct anthropogenic stressors. As such, bioassessment approaches used in the Arctic do not have the luxury of ignoring metrics or excluding the most abundant and diverse group of macroinvertebrates (Medeiros et al., 2011). As our analysis shows, increasing the taxonomic resolution of collected samples improves the sensitivity of biological metrics and may also overcome temporal variability, especially when considering trait‐based metrics in a warmer future.

## 5 CONCLUSION

As small lotic ecosystems in the Arctic are under‐represented in research, greater assessment of these systems is fundamentally important to understand the direct and indirect environmental stressors over time. Here, we establish that modified standard biomonitoring approaches are effective in detecting biological impairment from anthropogenic activity. The analyses of benthic invertebrates, as well as closer analyses of Chironomidae subfamilies, demonstrated selective exclusion of pollution‐sensitive taxa from impacted sites. The demonstrated shift in benthic invertebrate assemblages along a degradation gradient highlights the utility of biomonitoring of Arctic aquatic ecosystems, which will also increase our knowledge of how diversity may be influenced in a warming future. Climate change influences rapid ecological change, which can enhance interannual temporal variability of macroinvertebrate community structure. Increasing the taxonomic resolution of identification improved the sensitivity of biological metrics in our analysis, which may also reduce the effect of temporal variability. While this increases the cost of such programs, the naturally low diversity of Arctic streams may warrant the trade‐off between cost and information gained. Wider application of high‐resolution approaches for biomonitoring will also increase our baseline knowledge of the trajectory of Arctic lotic ecosystems, which will improve our ability to document change.

## AUTHOR CONTRIBUTION

**Andrew S Medeiros:** Conceptualization (lead); Data curation (supporting); Formal analysis (supporting); Funding acquisition (lead); Investigation (lead); Methodology (lead); Project administration (lead); Resources (lead); Supervision (lead); Validation (lead); Writing‐original draft (lead); Writing‐review & editing (lead). **Andrew Williams:** Data curation (supporting); Formal analysis (supporting); Investigation (supporting); Writing‐original draft (supporting). **Djuradj Milosevic:** Data curation (supporting); Formal analysis (lead); Investigation (supporting); Methodology (supporting); Project administration (supporting); Supervision (supporting); Writing‐original draft (supporting); Writing‐review & editing (supporting).

## Supporting information

Supplementary Material

## Data Availability

Raw abundance values of taxonomic data associated with this manuscript are archived at the Polar Data Catalogue, CCIN Reference #13209, https://doi.org/10.21963/13209.
