# Supplementary material for: Assessment of ecological impairment of Arctic streams: Challenges and future directions
Source: Ecol Evol. 2021 Jun 26;11(14):9715–27. doi: 10.1002/ece3.7798 (PMC8293736; doi:10.1002/ece3.7798)
Supplement: Supplementary file 1 — Supplementary Material [file ECE3-11-9715-s001.docx]

Supporting Information for:

**Assessment of ecological impairment of Arctic streams: challenges and future directions**

Medeiros A.S.^1*^, Williams A.^2^, Milošević D.^1,3^

1.School for Resource and Environmental Studies, Faculty of Management, Dalhousie University, Halifax, Canada

2.Department of Biology, Faculty of Science, Trent University, Peterborough, Canada

3.Department of Biology and Ecology, Faculty of Sciences and Mathematics, University of Niš, Niš, Serbia

**Contents of this file**

Text

Figures S1 to S6

Tables S1 to S2

**Introduction**

Snowmelt-dominated northern rivers and streams are under-represented in research (Docherty et al., 2019). As such, there are currently few aquatic monitoring programs or available data to accurately evaluate baseline conditions in an Arctic context (Medeiros et al., 2011). Thus, a crucial knowledge gaps exists with respect to how Arctic streams and rivers will be influenced by regional climate and local environmental stressors (Rouse et al., 1997; Medeiros et al., 2011; Kjikjerkovska, 2016). Aquatic ecosystems within Arctic watersheds are also generally reduced in size and extent, resulting in an increased sensitivity to environmental change due to a smaller reach and a lack of groundwater influence (Smith, 2018). Thus, these small Arctic watersheds can be early indicators of localized change with a rapid response per unit volume (Callaghan, 2011). Rouse et al. (1997) notes that small Arctic streams are often better representations of local catchment condition, not only because of smaller size, but due to their high reliance on terrestrial input from run-off; having a lower degree of autotrophic production. As such, monitoring of northern streams requires both baseline knowledge of northern environment-indicator relationships as well as an ability to elucidate change against the natural variability of these environments (Medeiros et al., 2011).

While Medeiros et al. (2011) found that adapted methodology was able to distinguish between polluted sites in the Arctic, questions of whether low taxonomic resolution of benthic invertebrates (e.g. to family level) potentially mask critical information necessary to distinguish temporal change remain. Here, we extend analysis of the community-based biomonitoring program first deployed by Medeiros et al. (2011) from 2007 to 2009 to include recent samples collected in 2014, 2015, 2018, and 2019 (Table S1). This supplementary information contains additional descriptions of the study area, additional methods, and additional results and interpretations from this study.

**Table S1** Sampling periods for biomonitoring program of the Apex River and Airport Creek in Iqaluit, Nunavut. Superscript letters stands for a) spatial-temporal matrix, b) seasonal-temporal matrix, and c) taxonomic scale (taxonomic sufficiency) matrix which are composed of data from particular campaigns.

|  |  | Period 1 | Period 2 | Period 3 |
| --- | --- | --- | --- | --- |
| Year | River | Early Summer | Mid Summer | Late summer |
| 2008 | Airport Creek |  | 07/17/2008^a^ |  |
|  | Apex River |  | 07/21/2008^a^ |  |
| 2009 | Airport Creek |  | 07/08/2009^a^ |  |
|  | Apex River |  | 07/10/2009^a^ |  |
| 2014 | Airport Creek | 7/4/2014^a^ | 8/6/2014^a^ |  |
|  | Apex River | 6/30/2014^a^ | 7/24/2014^a^ |  |
| 2015 | Airport Creek |  | 7/4/2015^a^ |  |
|  | Apex River |  | 7/6/2015^a^ |  |
| 2018 | Airport Creek | 7/2/2018^a,b^ | 7/10/2018^a,b^ | 8/4/2018^a,b^ |
|  | Apex River | 6/29/2018^a,b^ | 7/9/2018^a,b^ | 8/3/2018^a,b^ |
| 2019 | Airport Creek |  | 7/12/2019^a,c^ | 8/1/2019^a,c^ |
|  | Apex River |  | 7/10/2019^a,c^ | 7/31/2019^a,c^ |

**Study area**

Iqaluit, Nunavut, has a population of ~7,500 (Medeiros et al., 2011). The city rests on underlying terrain of Precambrian bedrock, with glacial till and veneer deposits (Harrison et al., 2011). Vegetation cover consists of low-growing shrubs, grasses, and forbs in low abundance (Medeiros et al., 2011). Average temperatures are -9.3 °C over a 20-year period, while summer temperatures (July-August) average approximately 7.5 °C (Bakaic and Medeiros, 2016). The 30-year average of annual precipitation (rainfall and snow) is 404 mm per annum (Bakaic and Medeiros, 2016).

Two streams were examined, Airport Creek and the Apex River. The headwater reach of both systems are typical Arctic nival streams. The lower reaches of both watercourses flow directly through the urban boundary. For the Airport Creek, multiple industrial and commercial activities contribute to known contamination, including: stream diversion for road construction, gravel operations, commercial/residential construction, an airport which routinely uses fuel and de-icings agents, multiple diesel pipelines that traverse the creek, an old decommissioned military landfill site, a metals dump, and sled dogs that are often tied-up along the banks. Visible along the stream reach (and often found within the stream itself) are caches of industrial waste, household garbage, abandoned vehicles, discarded oil and gas tanks, and a variety of abandoned building materials including several kitchen sinks (personal observations, 2005-2019). The creek has been subjected to a host of contamination issues within the catchment; several of these point-source locations could be contributing to the degradation of the watercourse over time.

Transport of materials to the stream regularly occurs during spring melt, including a network of small ephemeral streams flow from the old (decommissioned) landfill site into the main channel of the creek (Medeiros et al., 2011). A formerly operational commercial greenhouse along the mid-point of the creek was decommissioned in 2016. Construction is ongoing within the city, and few measures are being taken to limit sedimentation. Peramaki and Decker (2000) have reported elevated levels of bioavailable lead surrounding a former military base, old and current dumpsites and within the downtown core – the “grid”. Samuelson (1998) reports on soil samples taken by the Department of National Defense and the Iqaluit Leachate Monitoring Program, which found elevated levels of heavy metals such as lead and copper surrounding the old dump site, most of which exceeded EPA standards. Likewise, Dick et al. (2010) noted elevated concentrations of short- and medium-chained chlorinated paraffins downstream of these sites.

The Apex River has minimal known impacts along its reach. While the periphery of the urban boundary is near the river, its only source of direct urban influence is at the mouth of the river, which flows past the small residential area known as Apex. Vegetation along its reach consists of tundra forbs, wet sedge meadow, grasses and dwarf shrubs (Edwards and Treitz, 2017). The watershed is topographically complex, and subsurface (under ice) flow has been observed in the late season – potentially from ground ice (Lamoureux and Lafrenière, 2018). Local community members have used the watershed both for recreation and subsistence; where hunting, fishing, and direct consumption of river water occurs regularly. In the fall of 2018, water was diverted from the river to replenish stocks to the City of Iqaluit’s water supply reservoir – Lake Geraldine, and the river has been discussed numerous times as an ill-conceived replenishment source up until 2026 (NIRB, 2019).

**Additional Methods**

*For the creation of Figure 1:*

In order to delineate the extent of both watersheds, the 2m resolution Arctic Digital Elevation Model (Porter et al. 2018) was used in conjunction with CanVec geospatial data (NRCAN, 2015) to visualize the flow accumulation of both rivers in ArcMap 10.5. The area of each watershed was subsequently calculated in ArcMap. The urban boundary for Iqaluit was created through a 1000m buffer around all buildings in the CanVec data layer.

*For the analysis of invertebrate community structure:*

To analyze the differences in community structure between sampling locations, enumerated specimens (family/subfamily and genus, subgenus, and species level) were compared in a two-way crossed distance-based permutational multivariate analysis of variance (PERMANOVA; Clarke and Warwick, 2001) with a Bray–Curtis resemblance matrix using the Primer statistical package (v61.14). This approach focused on two factors, time (with 6 levels: 2008, 2009, 2014, 2015, 2018 and 2019) and space (with three levels, head, mid and mouth river sections), using 9999 permutations to estimate the p-value of pseudo-F statistic. Once the PERMANOVA models had been obtained, variability patterns of invertebrate community structure were visualized by a Principal Coordinates Analysis (PCoA). The same Bray-Curtis resemblance matrices were also used for PERMANOVA and applied for ordinating sampling sites onto PCoA axes.

**Results and interpretations**

We found similar results as Medeiros et al. (2011), in which a small Arctic stream that flows through a zone of heavy urban influence was found to have significantly lower diversity at sampling locations inside the urban zone compared to headwater locations. We also note that while development along a second river along the urban periphery (the Apex River) has occurred since 2009, there were few differences observed in either the abundance or diversity of benthic macroinvertebrates in any sampling year or period. Here we present additional results that support the interpretations and conclusions of the main paper. We also present the list of taxa used in our analysis (Table S2).


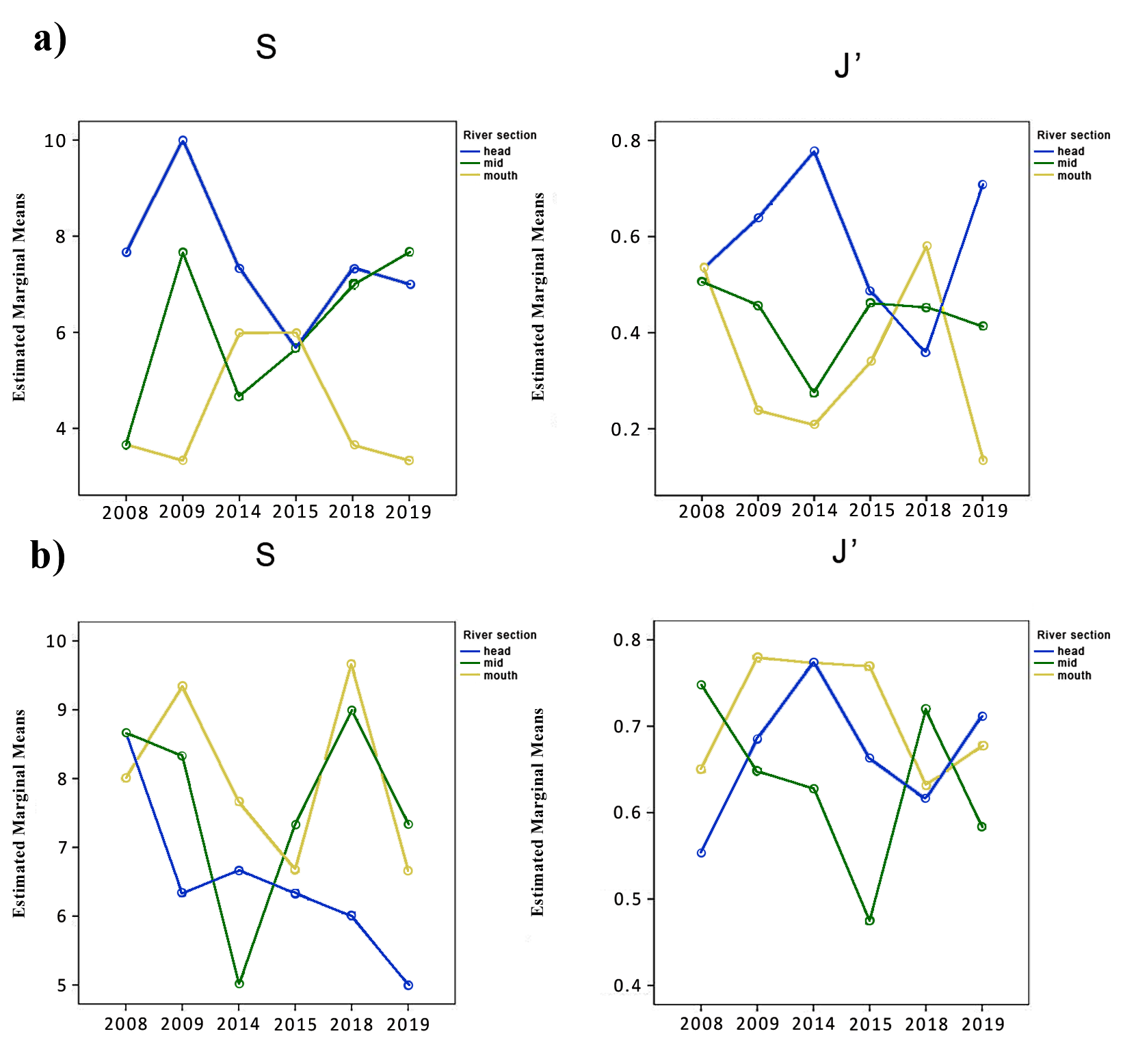


**Figure S1** Interaction between within- (time) and between- (river section) subject factors, presenting mean scores of diversity indices (Species richness (S), and Pielou evenness index (J’), for (a) Airport Creek and (b) Apex River.

**
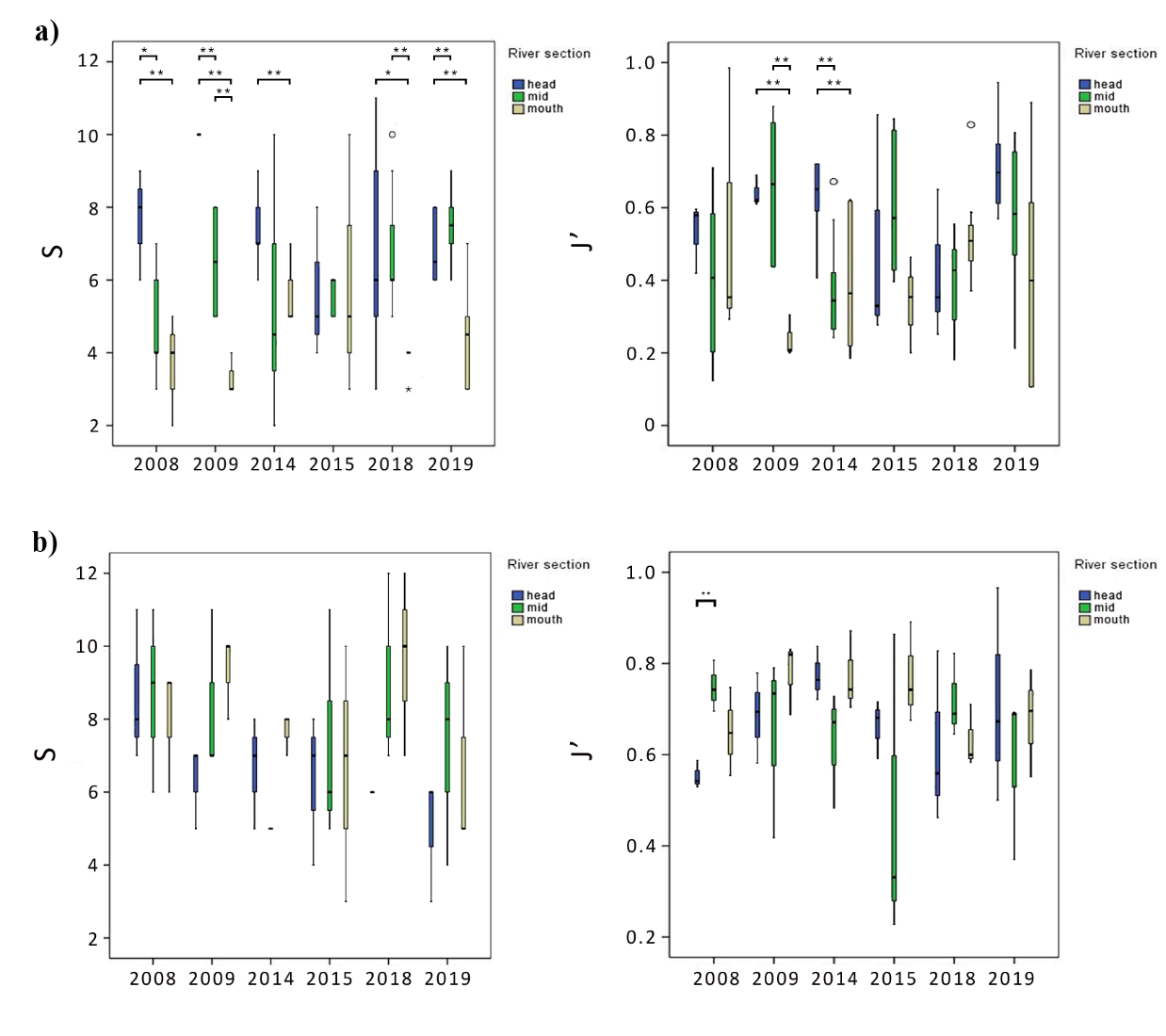
**

**Figure S2** Boxplot of diversity indices, Species richness (S), and Pielou evenness index (J’), across rivers section (head, mid, and mouth) and time (2014, 2015, 2018, and 2019) of (a) Airport Creek and (b) Apex River. Significant differences are noted (*p<0.05, **p<0.01).


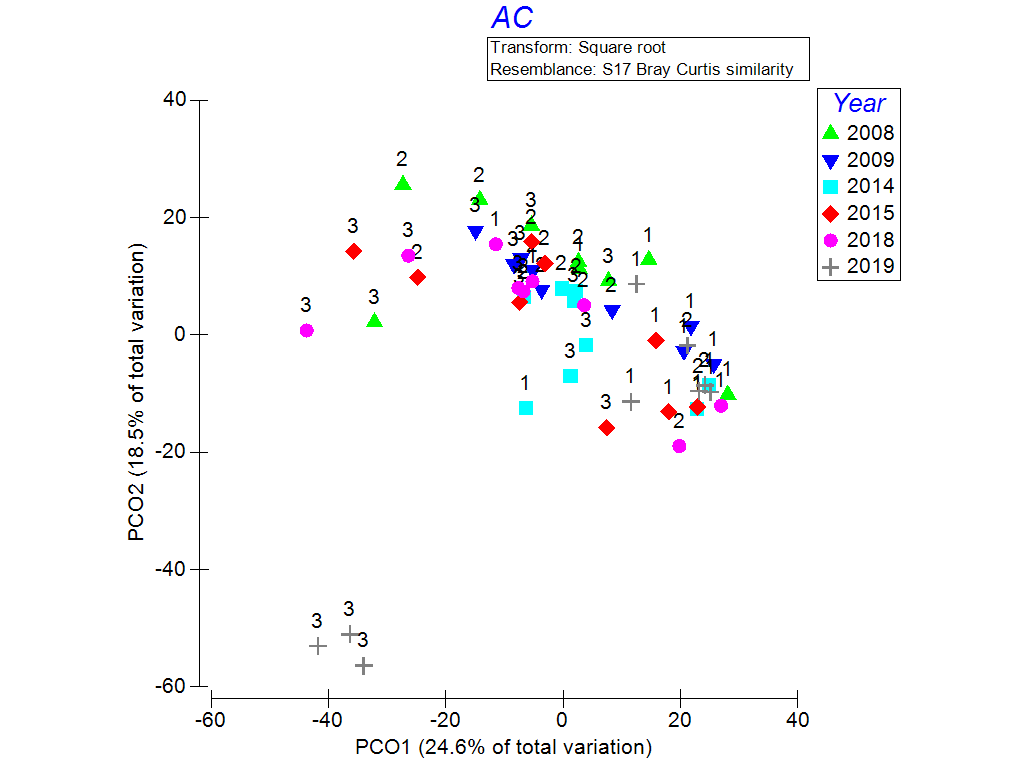


**Figure S3** Principal coordinates analysis (PCoA) scatterplot along the first two axes. Samples presented by different symbols and colors were sampled during different years (2014, 2015, 2018 and 2019) while different labels stand for particular rivers section of Airport Creek (AC): 1-head, 2-mid, and 3-mouth.


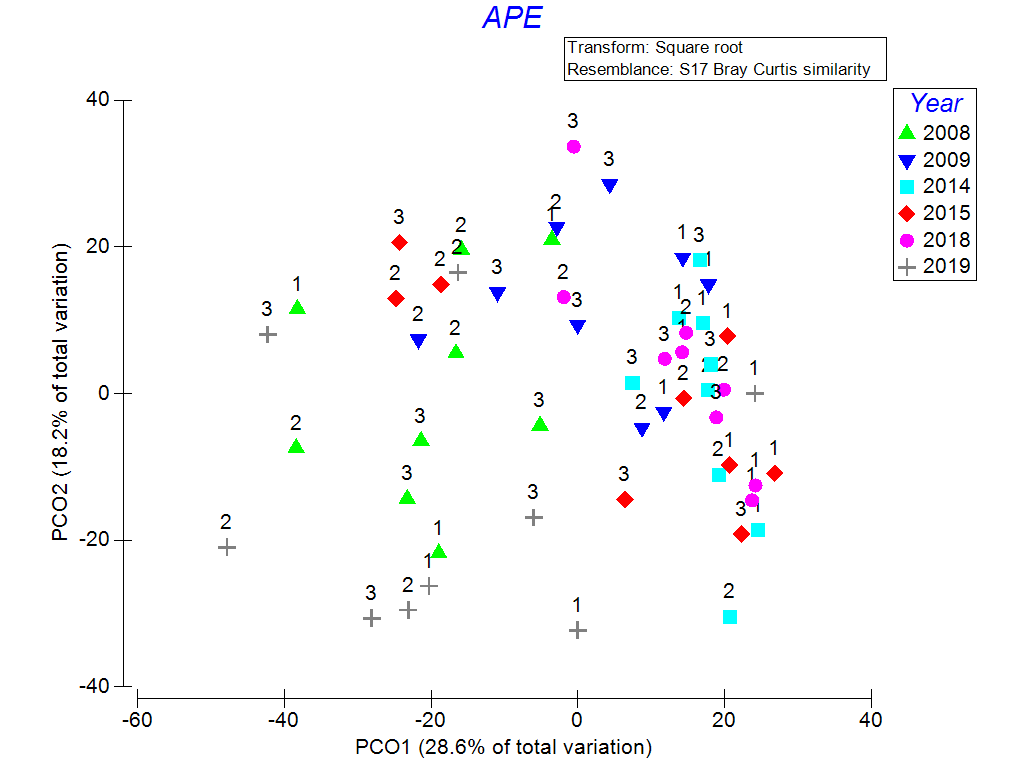


**Figure S4** Principal coordinates analysis (PCoA) scatterplot along the first two axes. Samples presented by different symbols and colors were sampled during different years (2014, 2015, 2018 and 2019) while different labels stand for particular rivers section of Apex River (APE): 1-head, 2-mid, and 3-mouth.


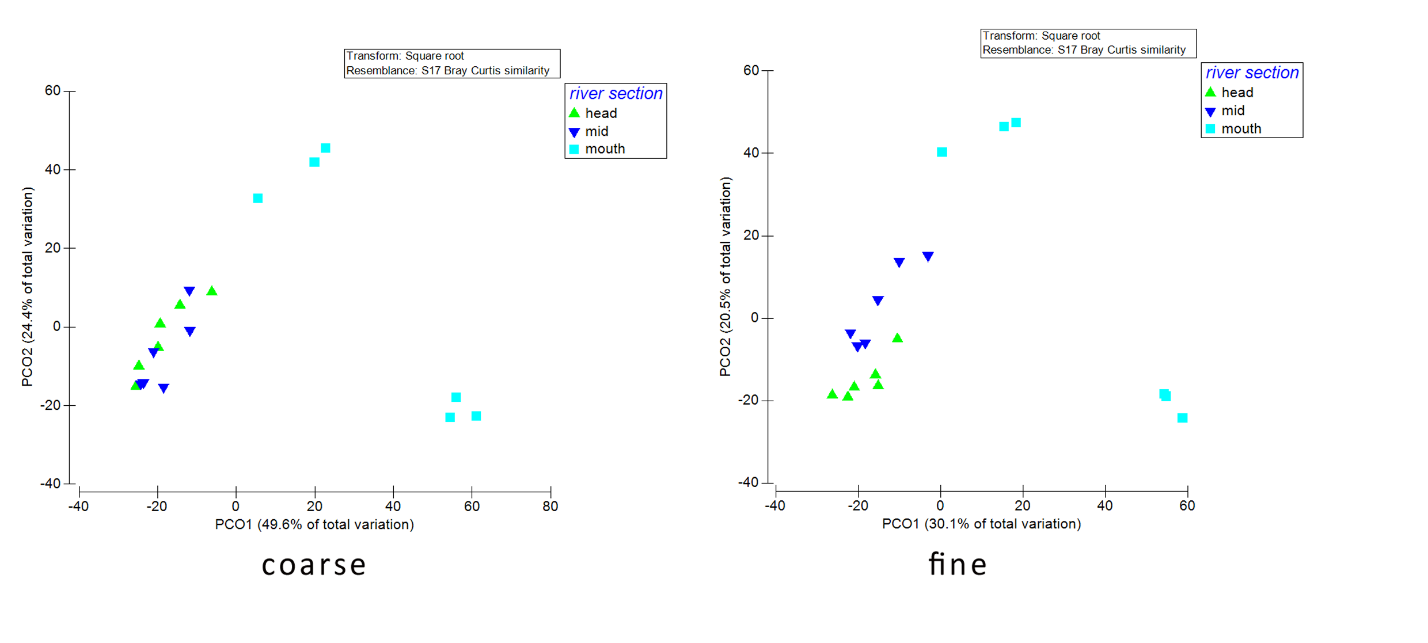


**Figure S5** Principal coordinates analysis (PCoA) scatterplot along the first two axes, based on community data of Airport Creek with coarse (left) and fine (right) taxonomic resolution. Samples presented by different symbols and colors were sampled during different river sections (head mid and mouth)

**
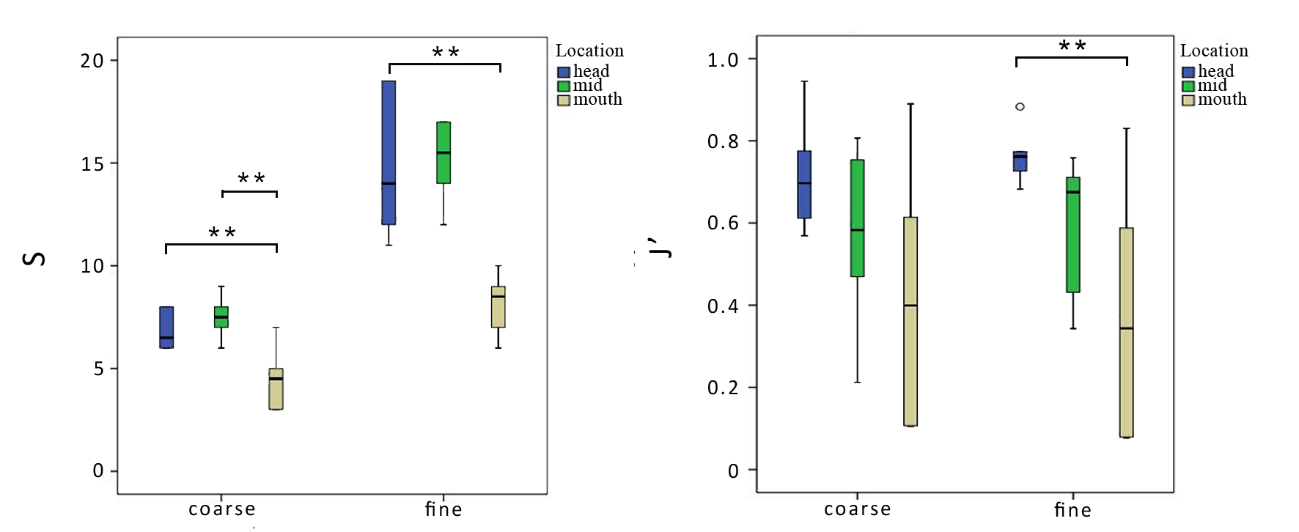
**

**Figure S6** Boxplot of diversity indices; Species richness (S), and Pielou evenness index (J’), based on the data sets with coarse (family/subfamily-level) and fine (species-level) taxonomic resolution for Airport Creek. LSD and Mann–Whitney post -hoc tests were applied for post-hoc comparison (*p<0.05, **p<0.01)

**Table S2** Taxonomic identification of collected macroinvertebrate specimens for the coarse resolution approach (e.g., Medeiros et al. 2011), and the highest possible taxonomic resolution (Fine Scale). The FFG presents feeding behavior and are assigned to one of 5 categories: gatherers/collector- (Cg), active filter feeder (F), predators (P), scraper (Sc), and shredders (Sh). Seven biological and ecological groups were used to describe the life-history traits (size, life-cycle, respiration, reproduction, and locomotion; Usseglio‐Polatera et al., 2000) and ecological requirements (distribution, favorable substrate, and current velocity; Usseglio‐Polatera et al., 2000), respectively.

| No | Coarse Scale | Fine Scale | |  |  |  |
| --- | --- | --- | --- | --- | --- | --- |
|  | Taxa | Taxonomic group | Taxa | FFG | BioT | EcoT |
| 1 | Tipulidae | Diptera (Chironomidae) | Allocladius bothnicus (Tuiskunen, 1984) | Sc | e | D |
| 2 | Hydrachnidae | Ephemeroptera (Ameletidae) | Ameletus sp. [Merritt and Cummins, 1996] | Sc | f | B |
| 3 | Plecoptera | Diptera (Chironomidae) | Arctopelopia sp [Andersen et al., 2013] | P | c | D |
| 4 | Oligochaeta | Ephemeroptera | Baetis sp. [ Merritt and Cummins, 1996] | Sc | e | C |
| 5 | Trichoptera | Diptera (Chironomidae) | Brillia cf modesta (Meigen, 1830) | Sh | e | D |
| 6 | Nematoda | Coleoptera | Carabidae [ Merritt and Cummins, 1996] | P | g | G |
| 7 | Ephemeroptera | Diptera (Chironomidae) | Chironomus dorsalis agg. sensu Vallenduuk (2017) | Cg | e | F |
| 8 | Empididae | Diptera (Chironomidae) | Corynoneura arctica (Kieffer, 1923) | Sc | e | D |
| 9 | Simuliidae | Diptera (Chironomidae) | Corynoneura sp.1 [Andersen et al., 2013] | Sc | e | D |
| 10 | Amphipoda | Diptera (Chironomidae) | Corynoneura sp2 [Andersen et al., 2013] | Sc | e | D |
| 11 | Hirudinea | Diptera (Chironomidae) | Cricotopus annulator complex sensu Epler (2001) | Sc | e | D |
| 12 | Coleoptera | Diptera (Chironomidae) | Cricotopus bicinctus (Meigen, 1818) | Sc | e | D |
| 13 | Orthocladiinae | Diptera (Chironomidae) | Cricotopus festivellus (Kieffer, 1906) | SC | e | D |
| 14 | Tanypodinae | Diptera (Chironomidae) | Cricotopus sylvestris gr sensu Moller Pillot (1984) | Sc | e | D |
| 15 | Tribe Chironomini | Diptera (Chironomidae) | Cricotopus sp1 [Andersen et al., 2013] | Sc | e | D |
| 16 | Tribe Tanytarsini | Diptera (Chironomidae) | Cricotopus tremulus group (Linnaeus, 1758) | SC | e | D |
| 17 | Podonominae | Diptera (Chironomidae) | Diamesa sp1 [Andersen et al., 2013] | Sc | e | D |
| 18 | Diamesinae | Diptera (Chironomidae) | Diplocladius cultriger (Kieffer, 1908) | Cg | e | D |
| 20 |  | Coleoptera | Ditiscidae larvae [ Merritt and Cummins, 1996] | P | g | G |
| 21 |  | Diptera (Chironomidae) | Eukiefferiella brehmi group (Gowin, 1943) | Sc | e | D |
| 22 |  | Diptera (Chironomidae) | Eukieferiella minor/fittkaui sensu Schmid (1993) | SC | e | D |
| 23 |  | Diptera (Chironomidae) | Euryhapsis cilium (Oliver, 1981) | Sh | e | D |
| 24 |  | Diptera (Chironomidae) | Heleniella sp1 (Saether, 1969) | Cg | e | D |
| 25 |  |  | Hydrachnidae | P | f | A |
| 26 |  | Diptera (Chironomidae) | Hydrobaenus conformis (Holmgren, 1869) | Cg | e | D |
| 27 |  | Diptera (Chironomidae) | Krenosmittia halvorseni (Cranston & Saether, 1986) | Cg | e | D |
| 28 |  | Trichoptera | Limnephilidae (Kolenati, 1848) [ Merritt and Cummins, 1996] | Sh | g | G |
| 29 |  | Diptera (Chironomidae) | Limnophyes sp. (Eaton, 1885) | Sc | e | D |
| 30 |  | Diptera (Chironomidae) | Metriocnemus eurynotus (Holmgren, 1883) | P | e | D |
| 31 |  | Diptera (Chironomidae) | Metriocnemus sp1[Andersen et al., 2013] | P | e | D |
| 32 |  | Diptera (Chironomidae) | Micropsectra sp [Andersen et al., 2013] | Cg | c | D |
| 33 |  | Nematoda | Nematoda |  |  |  |
| 34 |  | Annelida | Oligochaetal | Cg | h | F |
| 35 |  | Diptera (Chironomidae) | Oliveridia hugginsi (Kansas, 1987) | Sh | e | D |
| 36 |  | Diptera (Chironomidae) | Orthocadius sp. [Andersen et al., 2013] | Sc | e | D |
| 37 |  | Diptera (Chironomidae) | Orthocladius (Eudactilocladius) sp. [Andersen et al., 2013] | SC | e | D |
| 38 |  | Diptera (Chironomidae) | Orthocladius (Euorthocladius) clarkei (Soponis, 1977) | Sc | e | D |
| 39 |  | Diptera (Chironomidae) | Orthocladius (Euorthocladius) saxosus (Tokunaga, 1939) | Sc | e | D |
| 40 |  | Diptera (Chironomidae) | Orthocladius (Euorthocladius) sp1[Andersen et al., 2013] | Sc | e | D |
| 41 |  | Diptera (Chironomidae) | Orthocladius (Euorthocladius) sp2[Andersen et al., 2013] | Sc | e | D |
| 42 |  | Diptera (Chironomidae) | Orthocladius (Euorthocladius) thienemani (Kieffer, 1908) | Sc | e | D |
| 43 |  | Diptera (Chironomidae) | Orthocladius (Mesoorthocladius) sp. [Andersen et al., 2013] | Sc | e | D |
| 44 |  | Diptera (Chironomidae) | Orthocladius (Orthocladius) dorenus (Roback, 1957) | Sc | e | D |
| 45 |  | Diptera (Chironomidae) | Orthocladius (Orthocladius) oliveri (Soponis, 1977) | Sc | e | D |
| 46 |  | Diptera (Chironomidae) | Orthocladius (Orthocladius) robacki (Soponis, 1977) | SC | e | D |
| 47 |  | Diptera (Chironomidae) | Orthocladius (Orthocladius) sp.1 [Andersen et al., 2013] | Sc | e | D |
| 48 |  | Diptera (Chironomidae) | Orthocladius (Orthocladius) sp2 [Andersen et al., 2013] | Sc | e | D |
| 49 |  | Diptera (Chironomidae) | Paralimnophyes longiseta (Thienemann, 1914) | Cg | e | D |
| 50 |  | Diptera (Chironomidae) | Parametriocnemus sp. [Andersen et al., 2013] | Cg | e | D |
| 51 |  | Diptera (Chironomidae) | Paraphaenocladius sp. [Andersen et al., 2013] | Cg | e | D |
| 52 |  | Diptera (Chironomidae) | Paratanytarsus cf lauterborny sensu Moller Pillot (1984) | Sc | e | D |
| 53 |  | Plecoptera | Plecoptera undifferentiated [ Merritt and Cummins, 1996] | Sh | f | A |
| 54 |  | Diptera (Chironomidae) | Procladius sp. [Andersen et al., 2013] | P | c | D |
| 55 |  | Diptera (Chironomidae) | Psectrocladius (Allopsectrocladius) patypus | Sc | e | D |
| 56 |  | Diptera (Chironomidae) | Psectrocladius septentrionalis (Chernovskij, 1949) | Sc | e | D |
| 57 |  | Diptera (Chironomidae) | Pseudokiefferiella parva (Edwards, 1932) | Sc | e | D |
| 58 |  | Diptera (Chironomidae) | Psilometriocnemus sp. [Andersen et al., 2013] |  | e | D |
| 59 |  | Diptera | Simuliidae [ Merritt and Cummins, 1996] | F | e | B |
| 60 |  | Diptera (Chironomidae) | Stictochironomus sp [Andersen et al., 2013] | Cg | e | F |
| 61 |  | Diptera (Chironomidae) | Synorthocladius cf semivirens (Kieffer, 1909) | Cg | e | D |
| 62 |  | Diptera (Chironomidae) | Tanytarsus spp. [Andersen et al., 2013] | Cg | e | D |
| 63 |  | Diptera | Tipulidae [ Merritt and Cummins, 1996] | Sh | c | D |
| 64 |  | Trichoptera | Hydrophilidae [ Merritt and Cummins, 1996] | P | g | G |
| 65 |  | Diptera (Chironomidae) | Trichotanypus posticalis (Lundbeck, 1898) | Sh | e | A |
| 66 |  | Diptera (Chironomidae) | Tvetenia cf bavarica (Goetghebuer, 1934) | Sc | e | D |
| 67 |  | Diptera (Chironomidae) | Tvetenia discoloripes agg (Goetghebuer in Thienemann, 1936) | Sc | e | D |

**References**

Bakaic, M., & Medeiros, A. S. (2016). Vulnerability of northern water supply lakes to changing climate and demand. Arctic Science, 3(1), 1-16. https://doi.org/10.1139/as-2016-0029

Callaghan, T. V., Johansson, M., & Brown, R. D. (2011). Changing snow cover and its impacts Snow, Water, Ice and Permafrost in the Arctic (SWIPA): Climate Change and the Cryosphere. Oslo: Arctic Monitoring and Assessment Programme, 4-1.

Docherty, C. L., Dugdale, S. J., Milner, A. M., Abermann, J., Lund, M., & Hannah, D. M. (2019). Arctic river temperature dynamics in a changing climate. *River Research and Applications*, *35*(8), 1212-1227. https://doi.org/10.1002/rra.3537

Dick, T. A., Gallagher, C. P., & Tomy, G. T. (2010). Short-and medium-chain chlorinated paraffins in fish, water and soils from the Iqaluit, Nunavut (Canada), area. *World Review of Science, Technology and Sustainable Development*, *7*(4), 387-401. <https://doi.org/10.1504/WRSTSD.2010.032747>

Edwards, R., & Treitz, P. (2017). Vegetation greening trends at two sites in the Canadian Arctic: 1984–2015. Arctic, Antarctic, and Alpine Research, 49(4), 601-619. https://doi.org/10.1657/AAAR0016-075

Epler, J. H. (2001). Identification manual for the larval Chironomidae (Diptera) of North and South Carolina (p. 526). Crawford, South Carolina: John H. Epler.

Harrison, J C; St-Onge, M R; Petrov, O V; Strelnikov, S I; Lopatin, B G; Wilson, F H; Tella, S; Paul, D; Lynds, T; Shokalsky, S P; Hults, C K; Bergman, S; Jepsen, H F; Solli, A. (2011). Geological Survey of Canada, "A" Series Map 2159A

Kjikjerkovska, E. (2016). Long-term hydroclimatic change and interannual variability in water sources, Apex River (Iqaluit), Baffin Island, Nunavut (Doctoral dissertation, Queens University). <http://hdl.handle.net/1974/14085>

Lamoureux, S., and Lafrenière, M. (2018). Section II: Terrestrial Systems; Water Security and Quality in the Changing Arctic, pp 383., In: ArcticNet. Lemay, M., Gaden, A., and Barrette, C. (Eds.). Impacts of Environmental Change in the Canadian Coastal Arctic: A Compendium of Research Conducted During ArcticNet Phase IV (2015-2016).

Merritt, R.W. and Cummins, K.W. eds., 1996. An introduction to the aquatic insects of North America. Kendall Hunt

Moller Pillot H. De larven der Nederlandse Chironomiae (Diptera). 1A: Inleiding, Tanypodinae en Chironomini. Leiden: St. E.I.S Nederland, 1984a.

Moller Pillot H. De larven der Nederlandse Chironomiae (Diptera). 1B: Orthocladiinae sensu lato. Leiden: St. E.I.S Nederland, 1984b.

Natural Resources Canada (2015). "CanVec" in Natural Resources Canada Earth Sciences Sector Canada Centre for Mapping and Earth Observation and Centre for Topographic Information, Sherbrooke, QC, Canada: Centre for Topographic Information. <https://ftp.maps.canada.ca/pub/nrcan_rncan/vector/canvec/shp/>

Nunavut Impact Review Board (2019). City of Iqaluit: Apex Drinking Water Supply. Retrieved from [January, 2021]: <http://www.nirb.ca/project/125429>

Porter, C., Morin, P., Howat, I., Noh, M. J., Bates, B., Peterman, K., ... & Bojesen, M. (2018). ArcticDEM. Harvard Dataverse, V1, [January, 2021] https://doi.org/10.7910/DVN/OHHUKH

Peramaki, L. A., & Decker, J. F. (2000). Lead in soil and sediment in Iqaluit, Nunavut, Canada, and links with human health. Environmental monitoring and assessment, 63(2), 329-339. <https://doi.org/10.1023/A:1006253529308>

Rouse, W. R., Douglas, M. S., Hecky, R. E., Hershey, A. E., Kling, G. W., Lesack, L., ... & Smol, J. P. (1997). Effects of climate change on the freshwaters of arctic and subarctic North America. Hydrological processes, 11(8), 873-902. https://doi.org/10.1002/(SICI)1099-1085(19970630)11:8<873::AID-HYP510>3.0.CO;2-6

Samuelson, G. M. (1998). Water and waste management issues in the Canadian Arctic: Iqaluit, Baffin Island. Canadian Water Resources Journal, 23(4), 327-338. <https://doi.org/10.4296/cwrj2304327>

Smith, K. A. (2018). Snow Accumulation in the Niaqunguk (Apex) River Watershed near Iqualuit, Nunavut, Canada (Doctoral dissertation, Carleton University). <https://doi.org/10.22215/etd/2018-13218>

Usseglio‐Polatera, P., Bournaud, M., Richoux, P., & Tachet, H. (2000). Biological and ecological traits of benthic freshwater macroinvertebrates: relationships and definition of groups with similar traits. *Freshwater Biology*, 43(2), 175-205. <https://doi.org/10.1046/j.1365-2427.2000.00535.x>

Vallenduuk, H.J. 2017. Chironomini larvae of western European lowlands (Diptera: Chironomidae) Keys with notes to the species Lauterbornia.
